# Supplementary material for: A Formative Assessment of Antibiotic Dispensing/Prescribing Practices and Knowledge and Perceptions of Antimicrobial Resistance (AMR) among Healthcare Workers in Lahore Pakistan
Source: Antibiotics (Basel). 2022 Oct 16;11(10):1418. doi: 10.3390/antibiotics11101418 (PMC9598410; doi:10.3390/antibiotics11101418)
Supplement: Supplementary file 1 [file antibiotics-11-01418-s001.zip › antibiotics-1917565-supplementary.pdf]

Part 1: Demographics, Workplace Characteristics and Dispensing Practices

Record ID ______________________________

Demographics / ڈیموگرافی

Survey Date / سروے کی تاریخ ______________________________

Data Collector / سروے ڈیٹا کلیکٹر ______________________________

UC / یو سی ______________________________

Health Facility ID / ہیلتھ مرکز ______________________________

Age / عمر ______________________________

Gender / جنس
- ◯ Male
- ◯ Female
- ◯ Other
- ◯ Do not wish to disclose

What is your highest educational qualification? / آپ کی تعلیمی قابلیت کیا ہے؟(صرف ایک آپشن منتخب کریں-
- ☐ Primary school or less
- ☐ Secondary School
- ☐ Higher Secondary School FA/FSc
- ☐ University/college or Graduation/post-Graduation (4)
- ☐ Diploma Nursing
- ☐ BS Nursing
- ☐ MS Nursing
- ☐ Diploma Pharmacy
- ☐ BS Pharmacy
- ☐ MS/MPhil Pharmacy
- ☐ Doctor of Pharmacy
- ☐ MBBS
- ☐ Post-graduate medical degree
- ☐ Diploma Paramedic/Medical Technician
- ☐ Other (please list below)

Other professional qualification / '- دیگر تعلیمی قابلیت کی وضاحت کریں ______________________________

Year of completed education / کس سال میں آپ نے اپنی آخری تعلیمی قابلیت پوری کی؟ ______________________________

Place of Employment / ملازمت کی جگہ-	◯ Private Clinic (Group/Hospital)<br>◯ Private Clinic (Individual)<br>◯ Basic Health Unit (BHU)<br>◯ Taluka Hospital<br>◯ Pharmacy<br>◯ Medicine Shop
Type of Facility / صحت کی سہولیات فراہم کرنہ والہ آدارہ کی قسم	◯ For profit<br>◯ Not for profit
Level of Care / صحت کی سہولیات فراہم کرنہ والہ آدارہ کی نگہداشت کی سطح-	◯ Primary<br>◯ Secondary<br>◯ Tertiary
Years since facility is functioning/serving the community? / یہ ادارہ کب قائم کیا گیا ہے؟ کتنہسال سہ یہ ادارہ لوگوں کہ لیہ کام کر رہا ہہ؟	__________________________<br>(in years. if less than 1 year, use 1. if do not know, put 999.)
What is your current position? / آپ کی موجودہ پوزیشن کیا ہہ؟	◯ Physician<br>◯ Nurse BH/MN<br>◯ Lady Health Worker<br>◯ Lady Health Visitor (LHV)<br>◯ Midwife<br>◯ Pharmacist Manager<br>◯ Pharmacist Clerk<br>◯ Compounder/Dispenser<br>◯ Owner of the Medical shop<br>◯ Other (please specify below)
Other position / دیگر موجودہ پوزیشنز(اگر کوی ہہ-)	__________________________
How long have you served as a [current_position]? / آپ کتنہعرصہ سہ بطور [current_position] لوگوں کو سہولیات فراہم کر رہہ ہیں؟ کی خدمت کی ہہ (پوزیشن 1.9 کہ اوپر سوال میں بیان کی گئی ہہ)	__________________________<br>(in years. if less than 1 year, use 1. if do not know, put 999.)
How long have you been serving in your current position ([current_position]) at this facility? / آپ کتنہ عرصہ سہ اس ادارہ میں موجودہ پوزیشن پر صحت کی سہولیات فراہم کرہہ ہیں؟ (سال داخل کریں)	__________________________<br>(in years. if less than 1 year, use 1. if do not know, put 999.)

Workplace Demographics / کام کی جگہ

Number of persons employed at workplace / اس ادارہ میں کتنہ لوگ کام کرتہ ہیں؟(صرف وہ لوگ جو صحت کی سہولیات فراہم کر رہہ ہیں یا فارمیسی میں وہ لوگ جو ادویات خرید اور بیچ رہہ ہیں-)	__________________________<br>(if do not know, put 99999)
Average number of patients or clients per week / س اوسطاًایک ہفتہ میں کتنہ مریض یا گاہک آتہ ہیں	__________________________<br>(if do not know, put 99999)

Common infectious diseases among patients/clients (rank from 1 [most common] to 5 [least common]) / آپ ک□ مریضوں اور گا□کوں میں کون س□ عام امراض پا□ جات□ □یں؟ (1 س□ 5 تک درج□ بن کریں)

	1	2	3	4	5
Upper Respiratory Infection / اوپری سانس کا انفیکشن	◯	◯	◯	◯	◯
Gastrointestinal / معد□ کا انفیکشن	◯	◯	◯	◯	◯
UTI / پیشاب کی نالی کا انفیکشن	◯	◯	◯	◯	◯
Skin/Wound Infection / جلد/زخم کا انفیکشن	◯	◯	◯	◯	◯
PID / خواتین ک□ مخصوص امراض	◯	◯	◯	◯	◯

Dispensing Practices / ڈسپنس کرن□ ک□ طریق□

What percentage of your clients request antibiotics without a prescription? / آپ ک□ کتن□ فیصد مریض آپ س□ اینٹی بائیوٹکس مانگت□ □یں؟

- ◯ < 10%
- ◯ 11% to 25%
- ◯ 26% to 50%
- ◯ >50%

What percentage of your patients request antibiotics? / (غیر معالجین)آپ ک□ گا□کوں میں س□ کتن□ فیصد ڈاکٹر ک□ نسخ□ ک□ بغیر اینٹی بائیوٹکسزمانگت□ □یں؟؟

- ◯ < 10%
- ◯ 11% to 25%
- ◯ 26% to 50%
- ◯ >50%

Antibiotics commonly dispensed/prescribed (rank from 1 [most common] to 7 [least common] / عام طور پر تقسیم/تجویز کی جان□ والی اینٹی بائیوٹکس (درج□ بندی کریں 1 -انت□ائی عام س□ 7- کم از کم عام□

	Do not prescribe/ dispense	1	2	3	4	5	6	7
Amoxicillin/Augmentin / اموکسیلن/اگسٹین	◯	◯	◯	◯	◯	◯	◯	◯
Ampicillin / امپسلن	◯	◯	◯	◯	◯	◯	◯	◯
Azithromycin / ازیتھرومایسین	◯	◯	◯	◯	◯	◯	◯	◯
Cefixime / سیفکسائم	◯	◯	◯	◯	◯	◯	◯	◯
Ceftriaxone (injectable)	◯	◯	◯	◯	◯	◯	◯	◯
Cipro / سیپروفلوکسین	◯	◯	◯	◯	◯	◯	◯	◯
Co-trimoxazole	◯	◯	◯	◯	◯	◯	◯	◯

Are there any additional antibiotics that you prescribe/dispense on at least a weekly basis that are not listed above? / کیا کوئی اور ای اضافی اینٹی بائیوٹکس □یں جو آپ کم از کم □فت□ وار تجویز کرت□ □یں؟
(if do not prescribe/dispense, write "Not applicable")

Please answer the following questions:

How likely is it that you would prescribe an antibiotic for the following conditions? / (معالج) درج زیل حالات میں اس بات کا کتنا امکان □□ ک□ آپ اینٹی بائیوٹکس تجویز کریں؟

How likely is it that you would dispense an antibiotic for the following conditions without a physician's prescription? / (غیر معالج) کتنا امکان □□ ک□ آپ ڈاکٹر ک□ نسخ□ ک□ بغیر درج ذیل حالات میں اپن□ گا□کوں کو اینٹی بائیوٹکس دت□ [illegible]

	Would not give antibiotics	Not Likely	Likely	Very Likely
Fever in a child / بچوں میں بخار ب	○	○	○	○
Fever in an adult / بالغ افراد میں بخار	○	○	○	○
Cough in a child / بچوں میں کھانسی	○	○	○	○
Cough in an adult / بالغ افراد میں کھانسی	○	○	○	○
Sore throat in a child / بچوں میں گل□ کی سوزش	○	○	○	○
Sore throat in an adult / کو بالغ افراد میں گل□ کی سوزش	○	○	○	○
Diarrhea (lasting more than a day) in a child / بچ□ میں ایک دن س□ زیاد□ د اس□ال کی صورت میں	○	○	○	○
Diarrhea (lasting more than a day) in an adult / بالغ افراد میں ایک دن س□ زیاد اس□ال کی صورت میں	○	○	○	○
Burning sensation when urinating in an adult / الغ افراد میں پیشاب کرت□ وقت جلن میں	○	○	○	○
Redness and/or swelling around a wound / زخم ک□ گرد ور□میا سوز ش	○	○	○	○
Discharge from a wound / زخم س□ اخراج/پیپ آنا	○	○	○	○

I have sold to patients or clients a part of an antibiotic course because he/she did not have enough money to buy the entire course. / (فارماسسٹ/ڈاکٹر جواینٹی بائیوٹکس بیچت□□یں) کیا آپ ن□ کسی مریض یا گا□ک کو اینٹی بائیوٹک ک□کورس کا کچھ حص□ بیچا کیونک□ اس ک□ پاس اتن□ پیس□ ن□یں تھ□ ،ک□ و□ مکمل کورس جتن□ دن ڈاکٹر ن□ تجویز کیا، و□ خرید سک□ ؟

○ Yes
○ No

Part 2: Knowledge and Perceptions

Record ID ______________________________

Knowledge:

In this next section, we want to ask you some true/false questions about antibiotics and antibiotic resistance. /اگلے
حصے میں ہم آپ سے اینٹی بائیوٹک اور اینٹی بائیوٹک مزاحمت کے بارے میں کچھ صحیحی/غلط سوالات پوچھنا چاہتے
ہیں۔ آپ بتایں کہ یہ غلط یہ صحیح ہیں؟

Have you heard about antibiotic resistance (AMR)? /
کیا آپ نے اینٹی بائیوٹک
مزاحمت (AMR) کے بارے میں سنا ہے؟

- ◯ Yes
- ◯ No

If no to the previous question, read this statement: Antimicrobial resistance is the phenomenon whereby commonly used antibiotics lose their efficiency against disease causing organisims (pathogens). In some cases, these pathogens can become resistant to multiple antibiotics (multi-drug resistance – MDR). / اگر 4.0 کا جواب نہیں ہے تو یہ
بیان پڑھیں: اینٹی مائیکروبیل مزاحمت ایک ایسا ہے جس کے تحت عام طور پر استعمال ہونے والی اینٹی بائیوٹکس
بیماری پیدا کرنے والے اعضاء (پیتھوجینز) کے خلاف اپنی کارکردگی کھو دیتی ہیں۔ کچھ معاملات میں ، یہ
پیتھوجینز متعدد اینٹی بائیوٹکس کے خلاف مزاحم بن سکتے ہیں (کثیر ادویات کے خلاف مزاحمت-MDR)

Antibiotics have saved millions of lives. / اینٹی
بائیوٹکس نے لاکھوں جانیں
بچائی ہیں۔

- ◯ True
- ◯ False

Antibiotics are good for treating infections caused by viruses. / اینٹی بائیوٹکس, وائرس
کی وجہ سے ہونے والے انفیکشن کے
علاج کے لیے اچھی ہیں۔

- ◯ True
- ◯ False

Antibiotics kill bacteria that cause illness. /
اینٹی بائیوٹکس و بیماریاں
پیدا کرنے والے بیکٹیریا کا
خاتمہ کرتے ہیں۔

- ◯ True
- ◯ False

Antibiotics kill good bacteria that protect the body from infection. / اینٹی بائیوٹکس اچھے
بیکٹیریا کا خاتمہ کرتے ہیں جو
کہ جسم کو انفیکشن سے بچاتے
ہیں۔

- ◯ True
- ◯ False

Antibiotics can cure colds and flu. / اینٹی
بائیوٹکس زکام اور فلو کا علاج
کر سکتی ہیں۔

- ◯ True
- ◯ False

Antibiotics can be used to treat COVID-19. /
اینٹی بائیوٹکس کرونا وائرس
COVID-19 کے علاج   میں استعمال
کیے جا سکتے ہیں؟

- ◯ True
- ◯ False

It is safe to use antibiotics from family, friends, and others. / اپنے خاندان کے فرد
یا دوستوں   سے اینٹی
بائیوٹکس لے کر استعمال کرنا
محفوظ ہے؟

- ◯ True
- ◯ False

Some people have allergies to antibiotics. / کچھ لوگوں کو اینٹی بائیوٹکس س□ الرجی □وتی □□□

◯ True
◯ False

A person should only stop using an antibiotic after consulting the prescriber (physician). / اینٹی بائیوٹک کا استعمال صرف ڈاکٹر ک□ مشور□ ک□ مطابق بند کرنا چا□ی□

◯ True
◯ False

Antibiotic resistance (AMR) is not a concern because new antibiotics will be available in the future. / اینٹی بائیوٹک مزاحمت (ا□ ایم آر) مسلئ□ ن□یں □□ کیونک□ مستقبل میں نئی اینٹی بائیوٹکس دستیاب □وں گی□

◯ True
◯ False

Antibiotic resistance is a significant problem in Pakistan. / پاکستان میں اینٹی بائیوٹک مزاحمت ایک ا□م مسئل□ □□□

◯ True
◯ False

The majority of antibiotic use occurs in inpatient hospital settings in Pakistan. / پاکستان میں اینٹی بائیوٹک کا زیاد□ تر استعمال ، □سپتال میں داخل شد□مریضوں ک□علاج ک□ لی□ □وتا □□□

◯ True
◯ False

Are you familiar with the World Health Organization AWaRe (Access, Watch, Reserve) categories for antibiotics? / کیا آپ ورلڈ □یلتھ آرگنائزیشن کی اینٹی بائیوٹکس AWaRe (رسائی ، گھڑی ، ریزرو) درج□ بندی س□□ واقف □یں؟

◯ Yes
◯ No

Are you familiar with antibiotic prescribing/dispensing guidelines from the Punjab Healthcare Commission/Primary and Secondary Healthcare Department? / [صرف پرائیویٹ فارماسسٹ) کیا آپ پنجاب □یلتھ کیئر کمیشن/پرائمری اینڈ سیکنڈری □یلتھ کیئر ڈیپارٹمنٹ کی اینٹی بائیوٹک تجویز/ڈسپنس کرن□ کی □دایات س□ واقف □یں؟

◯ Yes
◯ No
◯ Not applicable

Is your shop/business registered as a pharmacy with the Punjab Healthcare Commission/Primary and Secondary Healthcare Department/Chief Drug Controllers Office? / [صرف پرائیویٹ فارماسسٹ) کیا آپ کی دکان/کاروبار، فارمیسی ک□ طور پر پنجاب □یلتھ کیئر کمیشن/پرائمری اینڈ سیکنڈری □یلتھ کیئر ڈیپارٹمنٹ/چیف ڈرگ کنٹرولرز آفس میں رجسٹرڈ □□؟

◯ Yes
◯ No

Do you have a digital record keeping system of all antibiotics sales? / [صرف پرائیویٹ فارماسسٹ) کیا آپ ک□ پاس تمام اینٹی بائیوٹکس کی فروخت کا ڈیجیٹل ریکارڈ رکھن□ کا نظام □□؟	◯ Yes<br>◯ No
What digital system are you using to track the dispensing/sales of antimicrobials? / [صرف پرائیویٹ فارماسسٹ) آپ اینٹی بائیوٹکس کی ڈسپنسنگ/سیلز کا ریکارڈ رکھن□ ک□ لی□ کون سا ڈیجیٹل سسٹم استعمال کر ر□□ □یں	◯ Excel sheet<br>◯ Giga solution<br>◯ Abu Zar<br>◯ Origa<br>◯ Other (please list below)
Other digital system:	__________________________

Perceptions (Severity)

	SD	D	A	SA
Antibiotic resistance affects my patients'/customers' health and well being. / اینٹی بائیوٹک مزاحمت میر□ مریضوں/صارفین کی صحت اور فلاح و ب□بودو متاثر کرتی □□□	◯	◯	◯	◯
Antibiotic resistance affects my ability to help my patients/clients recover from infectious diseases. / اینٹی بائیوٹک مزاحمت میری مریضوں/ صارفین کو متعدی امراض س□ صحت یاب □ون□ میںمدد کرن□ کی صلاحیت کو متاثر کرتی □□□	◯	◯	◯	◯
Antibiotic resistance increases the cost of health care. / اینٹی بائیوٹک مزاحمت ،صحت کی دیکھ بھال کی قیمت میں اضاف□ کرتی □□□	◯	◯	◯	◯
Antibiotic resistance could effect my families' health and well-being. / اینٹی بائیوٹک مزاحمت میر□ خاندان کی صحت اور فلاح و ب□بود کو متاثر کر سکتی □□□	◯	◯	◯	◯

Perceptions (Self-Efficacy) / تصورات (خود افادیت)

	SD	D	A	SA

I can change my antibiotic prescribing/dispensing practices based on government guidelines. / میں حکومتی □دایات کی بنیاد پر اپن□ اینٹی بائیوٹک تجویز/فرا□م کرن□ ک□ طریقوں کو تبدیل کر سکتا □وں□	○	○	○	○
I can explain to my patients/customers why they do NOT need an antibiotic in certain situations (e.g., a viral infection). / میں اپن□ مریضوں/گا□کوں کو بتا سکتا □وں ک□ ان□یں بعض حالات میں اینٹی بائیوٹک کی ضرورت کیوں ن□یں □وتی (مثال ک□ طور پر ، وائرل انفیکشن)	○	○	○	○
I can be an advocate for antibiotic stewardship with my peers and colleagues. / میں اپن□ ساتھیوں اور اپن□ ساتھ کام کرن□ والوں ک□ ساتھ اینٹی بائیوٹک سٹیوردشپ اک □مایتی بن سکتا □وں□	○	○	○	○

Perceptions (Response Efficacy and Cost) / تاثرات (رسپانس افادیت اور لاگت)

	SD	D	A	SA
I can contribute to decreasing antibiotic resistance by changing my prescription/dispensing practices. / میں اپن□، ادویات تجویز کرن□□/ڈسپنسنگ ک□ طریقوں کو تبدیل کرک□، اینٹی بائیوٹک مزاحمت کو کم کرن□ میں اپنا کردایر ادا کرسکتا □وں□	○	○	○	○
I will lose clients or patients if I decrease prescribing/dispensing antibiotics. / اگر میں ن□اینٹی بائیوٹکس تجویز/ ڈسپنس کرناکم کیں تو مجھ□ کاروبار میں نقصان □و سکتا □□□	○	○	○	○
The few antibiotics that I prescribe/dispense do not affect antibiotic resistance in Pakistan. / چند اینٹی بائیوٹکس جو میں تجویز کرتا □وں/دیتا □وں، اس س□ پاکستان میں اینٹی بائیوٹک مزاحمت پر کوئاثر ن□یں پڑتا □	○	○	○	○

I would be considered an irresponsible healthcare provider if I did not provide antibiotics to patients/customers when they request them. / اگر میں اپنےمریضوں/گاہکوں کو ان کی درخواست پر اینٹی بائیوٹکس فراہم نہ کروں تو میں ایک غیر ذمہ دارصحت کی سہولیات فراہم کرنے والا سمجھا جاؤں گا-	○	○	○	○

Perceptions (Structural) / ساختی

	SD	D	A	SA
It is Pakistan/Punjab government's responsibility to do something about antibiotic resistance. / یہ پاکستان/پنجاب حکومت کی ذمہ داری ہے کہ وہ اینٹی بائیو ٹک کی مزاحمت کو روکنے کے لیے اقدامات کریں۔	○	○	○	○
The Pakistan/Punjab government does not provide sufficient information to the public about antibiotic resistance. / پاکستان/پنجاب حکومت، عوام کو اینٹی بائیوٹک مزاحمت کے بارے میں مناسب معلومات فراہم نہیں کرتی۔	○	○	○	○
I have opportunities to learn more about antibiotic resistance and stewardship through trainings and educational materials that are available to me. / میرے پاس اینٹی بائیوٹک مزاحمت اور سٹیوردشپ کے بارے میں جاننے کے لیے ٹریننگ اور تعلیمی مواد کے مواقع دستیاب ہیں۔	○	○	○	○
Government regulations impact the way that I prescribe/dispense medications. / حکومتی قواعد و ضوابط میرے ا اینٹی بائیوٹک تجویز/ڈسپنس کرنے کےطریقے پر اثر ڈالتے ہیں۔	○	○	○	○

Part 3: Economic and Social Drivers of Dispensing Practices

Record ID ________________________________

General Financial Considerations / عمومی مالی معاملات□

During the past one month, what percentage of your monthly revenue came from antibiotics? / آپ کی پچھل□ ایک ما□ کی آمدنی کا کتنا فیصد حص□ اینٹی بائیوٹکس کی وج□ س□ آیا؟

- ◯ < 10%
- ◯ 11% to 25%
- ◯ 26% to 50%
- ◯ >50%
- ◯ Does not sell ABs
- ◯ Don't know

What portion of your dispensing is from antibiotics?

- ◯ < 10%
- ◯ 11% to 25%
- ◯ 26% to 50%
- ◯ >50%
- ◯ Does not sell ABs
- ◯ Don't know

What percentage of your monthly profits comes from antibiotics? / . آپ ک□ ما□ان□ منافع کا کتنا فیصد حص□ اینٹی بائیوٹکس س□ آتا □□؟

- ◯ < 10%
- ◯ 11% to 25%
- ◯ 26% to 50%
- ◯ >50%
- ◯ Does not sell ABs
- ◯ Don't know

Demand Pressures / . ڈیمانڈ پریشر

We would now like to ask a few questions about hypothetical scenarios. How likely is it that you would provide antibiotics to a client/patient under the following scenarios? / اب □م فرضی صورتحال /منظرک□ بار□ میں چند سوالات پوچھنا چا□یں گ□□ کتنا امکان □□ ک□ آپ گا□ک /مریض کو مندرج□ ذیل صورتحال /منظر میں اینٹی بائیوٹکس فرا□م/ تجویز کریں گ□؟

An adult patient/client comes to the clinic/pharmacy and says, “I have had a fever and sore throat for two days. Can you sell me some medicine?”

ایک بالغ مریض/کلائنٹ، کلینک/فارمیسی میں آتا □□ اور ک□تا □□ ، "مجھ□ دو دن س□ بخار اور گل□ کی سوزش □□□ "کیا آپ مجھ□ کوئی دوا فرا□م / تجویز کر سکت□ □یں؟

- ◯ Would not give antibiotics
- ◯ Not likely
- ◯ Likely
- ◯ Very likely

An adult brings a child to the clinic/pharmacy and says, “He has had a fever and sore throat for two days. Can you sell us some medicine?”

ایک بالغ ایک بچ□ کو
کلینک/فارمیسی پر لاتا □□ اور
ک□تا □□ ک□، "اس□ دو دن س□ بخار
اور گل□ کی سوزش □□ □" کیا آپ
اس□ ین کوئی دوا فرا□م / تجویز
کر سکت□ □یں؟

- ◯ Would not give antibiotics
- ◯ Not likely
- ◯ Likely
- ◯ Very likely

An adult patient/client comes to the clinic/pharmacy and says, “I have had a fever and sore throat for two days. Can you sell me antibiotics?”

ایک بالغ مریض/کلائنٹ،
کلینک/فارمیسی میں آتا □□ اور
ک□تا □□ ، "مجھ□ دو دن س□ بخار
اور گل□ کی سوزش □□□" کیا آپ
مجھ□ کوئی اینٹی بائیوٹک
فرا□م / تجویز کر سکت□ □یں؟

- ◯ Would not give antibiotics
- ◯ Not likely
- ◯ Likely
- ◯ Very likely

An adult brings a child to the clinic/pharmacy and says, “He has had a fever and sore throat for two days. Can you sell us antibiotics?”

ایک بالغ ایک بچ□ کو
کلینک/فارمیسی پر لاتا □□ اور
ک□تا □□ ک□، "اس□ دو دن س□ بخار
اور گل□ کی سوزش □□ □" کیا آپ
اس□ کوئی اینٹی بائیوٹک فرا□م
/ تجویز کر سکت□ □یں؟ "

- ◯ Would not give antibiotics
- ◯ Not likely
- ◯ Likely
- ◯ Very likely

Demand Pressures / . ڈیمانڈ پریشر

We would now like to ask a few questions about hypothetical scenarios. How likely is it that you would provide antibiotics to a client/patient under the following scenarios? / اب □م فرضی صورتحال /منظرک□ بار□ میں چند سوالات پوچھنا چا□یں گ□□ کتنا امکان □□ ک□ آپ گا□ک /مریض ک
مندرج□ ذیل صورتحال /منظر میں اینٹی بائیوٹکس فرا□م/ تجویز کریں گ□؟

	Would not give antibiotics	Not Likely	Likely	Very Likely
An adult patient/client comes to the clinic/pharmacy and says, "I have had a fever and sore throat for two days. Can you [prescribe/dispense] me some medicine?"	◯	◯	◯	◯

An adult brings a child to the clinic/pharmacy and says, "He has had a fever and sore throat for two days. Can you [prescribe/dispense] us some medicine?"	○	○	○	○
An adult patient/client comes to the clinic/pharmacy and says, "I have had a fever and sore throat for two days. Can you [prescribe/dispense] me antibiotics?"	○	○	○	○
An adult brings a child to the clinic/pharmacy and says, "He has had a fever and sore throat for two days. Can you [prescribe/dispense] us antibiotics?"	○	○	○	○

Social Considerations:

We would now like to ask a few questions about what matters to you as a pharmacist and about your customers.

Rank the following in terms of how much they matter to you on your work as a pharmacist (rank from 1 [matters the most] to 4 [matters the least].

	1	2	3	4	N/A
Revenue/profit the facility makes from medication / فارمیسی کی آمدنی/منافع	○	○	○	○	○
My customers' satisfaction with the medicine I give them, and whether they seem me as a good [current_position] / میر□ گا□کوں کا اطمینان ان ادویات پر جو میں ان□یں دیتا □وں ، اور ی□ ک□ و□ مجھ□ ایک اچھ□ فارماسسٹ ک□ طور پر دیکھیں□	○	○	○	○	○
The extent to which my work complies with the government's regulations / و□ حد ج□اں تک میرا کام حکومت ک□ قواعد و ضوابط کی مطابق □□□	○	○	○	○	○

My reputation as a good [current_position] among my counterparts / میری ساکھ ،دیگر فارمیسسٹ میں ، بطور اچھ□ فارماسسٹ □ون□ کی □	○	○	○	○	○

AMR Trainings/IEC

Have you received any trainings or IEC materials about antibiotic resistance? / کیا آپ کو اینٹی بائیوٹک مزاحمت ک□ بار□ میں کوئی تربیت یا IEC مواد ملا □□؟

○ Yes
○ No

When was the most recent AMR training/material provided?

____
(write year of most recent training)

Were the trainings of IEC materials useful to increase your understanding of AMR? / کیا IEC مواد / تربیت ،آپ ک□ لی□ مفید تھی؟

○ Yes
○ No

Who provided you the antimicrobial resistance trainings/IEC material? / آپ کو اینٹی بائیوٹک مزاحمت کی تربیت/IEC مواد کس ن□ فرا□م کیا؟

____

What would be the best medium to provide you with information about antimicrobial resistance? / آپ کو اینٹی بائیوٹک مزاحمت ک□ بار□ میں معلومات فرا□م کرن□ کا ب□ترین ذریع□ کیا □وگا؟

____
